# Colorectal Cancers from Distinct Ancestral Populations Show Variations in *BRAF* Mutation Frequency

**DOI:** 10.1371/journal.pone.0074950

**Published:** 2013-09-16

**Authors:** Megan C. Hanna, Christina Go, Christine Roden, Robert T. Jones, Panisa Pochanard, Ahmed Yasir Javed, Awais Javed, Chandrani Mondal, Emanuele Palescandolo, Paul Van Hummelen, Charles Hatton, Adam J. Bass, Sung Min Chun, Deuk Chae Na, Tae-Im Kim, Se Jin Jang, Raymond U. Osarogiagbon, William C. Hahn, Matthew Meyerson, Levi A. Garraway, Laura E. MacConaill

**Affiliations:** 1 Center for Cancer Genome Discovery, Dana-Farber Cancer Institute and Harvard Medical School, Boston, Massachusetts, United States of America; 2 Department of Medical Oncology, Dana-Farber Cancer Institute and Harvard Medical School, Boston, Massachusetts, United States of America; 3 The Broad Institute, Cambridge, Massachusetts, United States of America; 4 Boston Baskin Cancer Foundation, Baptist Cancer Center, Memphis, Tennessee, United States of America; 5 Department of Pathology, University of Ulsan College of Medicine, Asan Medical Center, Seoul, South Korea; MOE Key Laboratory of Environment and Health, School of Public Health, Tongji Medical College, Huazhong University of Science and Technology, China

## Abstract

It has been demonstrated for some cancers that the frequency of somatic oncogenic mutations may vary in ancestral populations. To determine whether key driver alterations might occur at different frequencies in colorectal cancer, we applied a high-throughput genotyping platform (OncoMap) to query 385 mutations across 33 known cancer genes in colorectal cancer DNA from 83 Asian, 149 Black and 195 White patients. We found that Asian patients had fewer canonical oncogenic mutations in the genes tested (60% vs Black 79% (P = 0.011) and White 77% (P = 0.015)), and that *BRAF* mutations occurred at a higher frequency in White patients (17% vs Asian 4% (P = 0.004) and Black 7% (P = 0.014)). These results suggest that the use of genomic approaches to elucidate the different ancestral determinants harbored by patient populations may help to more precisely and effectively treat colorectal cancer.

## Introduction

It is well established that cancer is a genomic disease and many molecular and genetic alterations are well characterized. For example, somatic mutations in lung cancer [1], amplifications in breast cancer [2], and fusions in acute myeloid leukemia [3] are well known for their roles in both tumor biology and clinical behavior and outcome. We and others have previously shown that the frequency of somatic oncogenic mutations can vary across distinct ancestral populations. In non-small cell lung cancer (NSCLC), mutations in epidermal growth factor receptor (EGFR) are more prevalent in Asian patients compared to European-derived populations (8% versus 30%, P < 0.001). These mutations are also found more commonly in females and non-smokers [1,4]. Conversely, serine threonine kinase 11 (STK11) mutations (point mutations and deletions) are found in 17% of White NSCLC patients [5,6] but at much lower frequency in those from Asian patients. These observations raise the possibility that tumors from different ancestral groups may harbor distinct patterns of driver genetic alterations. Given that tumor somatic mutations can be both driver events and targets for effective pharmacological inhibition, knowledge of the associated ancestral determinants may have important implications for studies of both cancer health disparities and precision cancer medicine.

Colorectal cancer is the second leading cause of cancer death in the United States. As with several cancer types, colorectal cancer shows significant variation in incidence and mortality rates across Asian, Black and White populations. Blacks have the highest mortality rate (49.6 per 100,000) and Asians have the lowest (22.7 per 100,000) (US, SEER 2005-2009). Disparities in cancer outcomes between patients from different ancestral backgrounds have been demonstrated for many cancers, yet the mechanisms driving these disparities have not been clearly elucidated.

In the US, rates of incidence and mortality in colorectal cancer have been steadily declining due to increases in screening [7] with the largest declines for Whites and the smallest for Blacks. While variance in health care access and treatment patterns may play important roles, many cancers still exhibit differences even after accounting for these factors. For example, a 2004 study [8] found that Black patients were 1.67 times more likely to die of colorectal cancer at 5 years post-treatment than White patients.

In Korea, where incidence is still lower than in the US, the picture is complex with increasing rates of both incidence and survival [9]. Looking at global statistics, the rates of colorectal cancer have been increasing dramatically in many Asian countries with some experiencing a 2-4-fold increase in incidence of colorectal cancer, and some countries approaching rates of incidence similar to Western countries [10,11,12]. These increases in incidence are generally considered to result from the adoption of a more Western diet [10,12].

The overall 5-year survival rate for colorectal cancer is 64% in the U.S. (US, SEER 2005-2009). The range of stage specific rates is wide. Stage 1 tumors, which have grown into the colon but not extended outside of it, have an average 5-year survival of 89%. In contrast, those patients with distant metastasis have an average 5-year survival of 11%. Standard treatment for colorectal cancer consists of surgical resection for lower stage disease, and some combination of surgery, chemotherapy, and radiation for later stage. Refractory disease with distant metastasis, if *EGFR* expressing and *KRAS* wild type, can be treated with anti-*EGFR* monoclonal antibodies [13]. *KRAS* mutations at codons 12 and 13 are currently significant predictors of resistance to anti-*EGFR* monoclonal antibodies, and are of clinical utility in screening patients with metastatic disease to appropriate therapy [13].

To begin to assess the extent to which prevalent driver somatic mutations might vary with colorectal cancer patient ancestry [14,15], we conducted a systematic interrogation of 385 mutations in 33 known cancer genes in colorectal adenocarcinoma DNA from 427 patients including 83 Asian, 149 Black and 195 White patients by self-reported ancestry.

## Materials and Methods

### Patients and Tumor Specimen Collection

Our work has been reviewed by the Dana-Farber Cancer Institute Internal Review Board (DFCI IRB). We requested and were granted an exemption from DFCI IRB review as all specimens in the study were de-identified and anonymized.

We analyzed 427 tissue specimens (426 paraffin embedded and 1 fresh frozen) from patients with colorectal cancer. The anonymized tumor specimens were obtained from the Cooperative Human Tissue Network (CHTN), the Pathology Specimen Locator of Dana Farber/Harvard Cancer Center (DF/HCC), the University of Tennessee Health Science Center (UTHSC) and Asan Medical Center. DFCI IRB exemption was obtained for all samples. Limited pathology information was included for each specimen with race as a requirement and most frequently including gender, age, stage, location, and node or metastasis status.

For all specimens, tissue was sectioned and hematoxylin and eosin (H&E)-stained slides were obtained. Tumor-enriched areas were identified and core punches were taken from the corresponding region. Required tumor content was 70%. Accompanying biopsy diagnoses (obtained from CHTN, DF/HCC, UTHSC or Asan) were confirmed by independent histopathological review. DNA was extracted from FFPE cores using a QiaCube with the Qiagen QIAamp DNA FFPE Tissue Kit and from frozen specimens using either the Qiagen QIAamp or the Qiagen DNeasy kit according to the manufacturer’s directions.

### OncoMap genotyping

OncoMap (version 3) is a mass-spectrometric genotyping platform that assays for 385 mutations in 33 cancer genes listed in Table 1. Selection of cancer gene mutations for assay design and mass spectrometric genotyping were performed as previously described [14,15].

**Table 1:** OncoMapV3.

**Gene**	**Mutations**
ABL1	14
AKT1	1
AKT2	2
APC	12
BRAF	43
CDK4	1
CDKN2A	10
CSF1R	6
CTNNB1	31
EGFR	44
ERBB2	6
FGFR1	2
FGFR2	6
FGFR3	8
FLT3	8
HRAS	12
JAK2	1
JAK3	3
KIT	25
KRAS	22
MET	6
MLH1	1
MYC	6
NRAS	19
PDGFRA	17
PIK3CA	14
PTEN	14
RB1	11
RET	13
SRC	1
STK11	12
TP53	7
VHL	7
**33**	**385**

DNA quality was evaluated by quantification using Quant-iT™ Pico Green® dsDNAassay Kit (Invitrogen) per manufacturer’s protocol. 100ng genomic DNA was subjected to whole genome amplification (WGA) using either the Sigma-Aldrich GenomePlex Complete Whole Genome Amplification Kit for fresh frozen or the Qiagen RepliG Mini Kit for FFPE. IPLEX chemistry was used to generate a list of candidate mutations. Unamplified genomic DNA was used to validate all candidate mutations using a second (hME) chemistry. All methods were performed as previously described [14].

### Statistical analysis

Statistical significance was ascertained with a pair-wise comparison using a two-tailed Fisher’s Exact Test with a Bonferroni correction for multiple comparisons and a cut-off of less than 0.05.

## Results

### Patient characteristics

Colorectal cancer DNA from 83 Asian, 149 Black and 195 White patients (Table 2) was analyzed using the OncoMap platform The average patient age in our cohort was 65 years (Asian, 63; Black, 62; White, 68) and there were more women than men (224 vs 197). Ancestry was determined by self-reporting. In our cohort we found colorectal cancers to be most frequently located in the proximal colon (49%), and more commonly Stage 3 (55%). Proximal colorectal cancers were more common in White patients compared to Black (P = 0.009) and Asian (P = 3.69E-07) patients. Distal cancers were most common in Asians (P = 0.005 compared to Whites). Distal cancers were more common in females (P = 0.007), whereas proximal cancer was more common in males (P = 0.019). The Asian cohort had significantly more stage I and II disease compared to both Black (stage I, P = 0.002, stage II, P = 0.01) and White (stage I, P = 3.08E-06, stage II, P = 3.37-05) patients. White patients had more stage IV disease compared to Asians in this cohort (P = 0.004). Looking at the overall average staging for all groups, we found Whites to have the highest average stage of disease and Asians the lowest (Asian, 2.41; Black, 2.89; White, 3.09).

**Table 2:** Patient characteristics.

		**Asian**	**Black**	**White**	**Total**
**Gender**	*Female*	35	87	102	224
	*Male*	47	59	91	197
	*unknown*	1	3	2	6
		***83***	***149***	***195***	***427***
**Age (mean**)	*Female*	62 (33-87)	62 (30-90)	68 (36-90)	65 (30-90)
	*Male*	64 (43-84)	62 (26-87)	66 (22-91)	64 (22-91)
**Stage**	*Stage-I*	11 (13%)	3 (2%)		14 (3%)
	*Stage-II*	28 (34)	26 (17%)	21 (11%)	75 (18%)
	*Stage-III*	36 (43%)	87 (58%)	113 (58%)	236 (55%)
	*Stage-IV*	4 (5%)	19 (13%)	38 (19%)	60 (14%)
	*unknown*	4 (5%)	15 (10%)	23 (12%)	42 (10%)
**Location**	*distal*	33 (39%)	37 (25%)	41 (21%)	111 (26%)
	*proximal*	22 (27%)	67 (45%)	119 (61%)	208 (49%)
	*rectal*	25 (30%)	25 (17%)	19 (10%)	69 (16%)
	*unspecified*	3 (4%)	20 (13%)	16 (8%)	39 (9%)

### Cancer gene mutations in colorectal cancer

**Figure 1:**
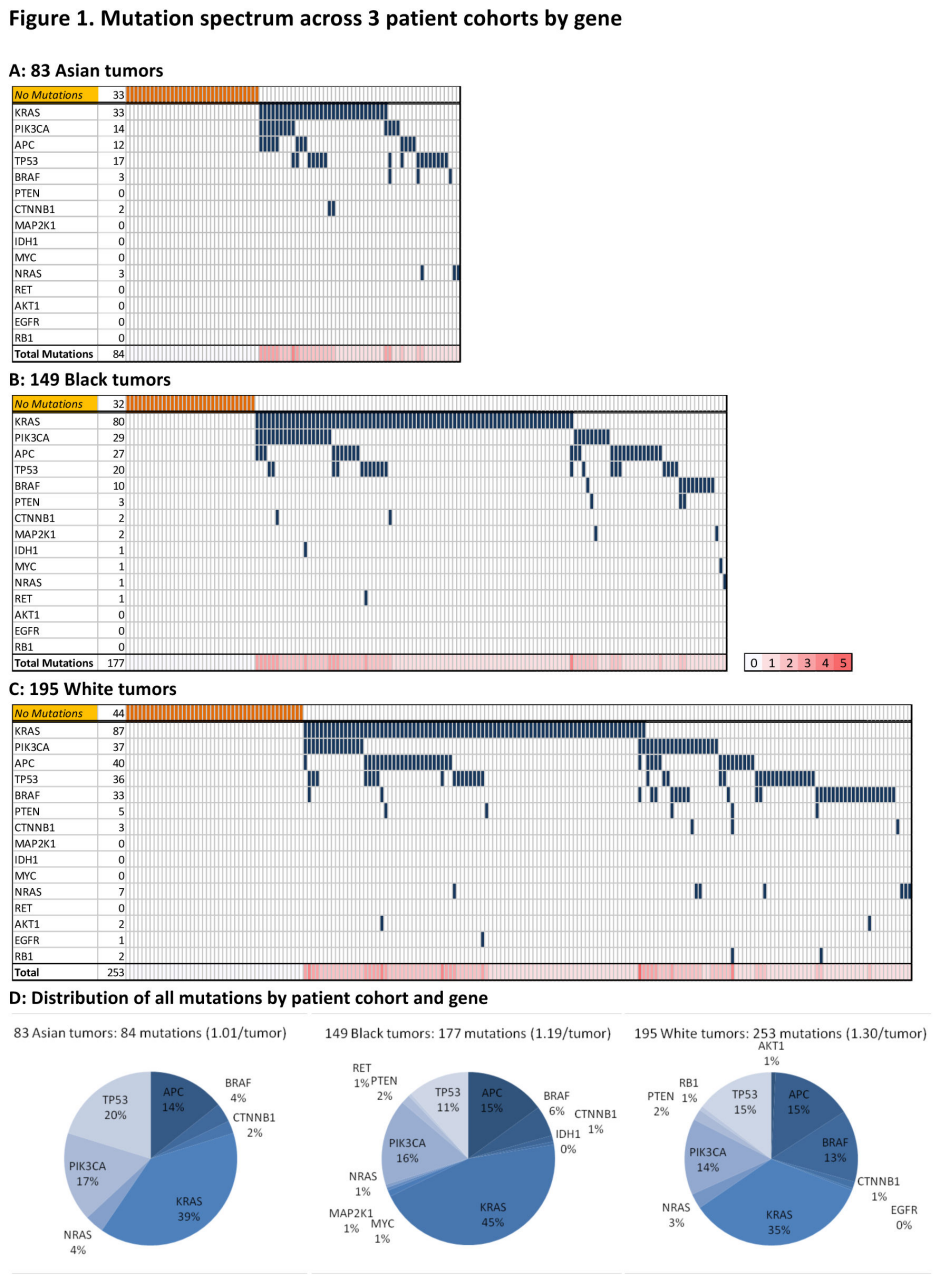
Mutation spectrum by tumor and gene. A summary chart of all mutations in each group displays genes across in rows and patient tumors down in columns. The bottom row of each represents the total number of mutations per tumor as a heat map (0-5 mutations). Chart A displays Asian patients, chart B, Black patients and chart C, White patients. Chart D displays the distribution of all mutations for each cohort.

Using OncoMap, we identified 514 mutations in 427 colorectal cancer specimens (Figure 1). We found 84 mutations in 83 tumors from Asian patients, 177 mutations in tumors from 149 Black patients and 253 mutations in 195 tumors from White patients. 318 tumors (74%) harbored at least one mutation. Of the tumors with mutations, 173 (40%) had single mutations and 145 (34%) had more than one mutation. Seven samples harbored 4 or more mutations. Mutations in *APC* (18%), *BRAF* (11%), *KRAS* (46%), *PIK3CA* (18%) and *TP53* (17%) were found most frequently (Table 3). We found the mutation rates of common oncogenes to be at frequencies similar to those previously reported for colorectal cancer [16].

**Table 3:** Gene mutations per patient population.

**Genes**	**Asian (83**)	**%**	**Black (149**)	**%**	**White** (195)	**%**	**Total (427**)	**%**
AKT1					2	1%	2	0.5%
APC	12	14%	26	17%	37	19%	75	17.6%
BRAF	3	4%	10	7%	33	17%	46	10.8%
CTNNB1	2	2%	2	1%	3	2%	7	1.6%
EGFR					1	1%	1	0.2%
IDH1			1	1%		0%	1	0.2%
KRAS	32	39%	79	53%	85	44%	196	45.9%
MAP2K1			2	1%		0%	2	0.5%
MYC			1	1%		0%	1	0.2%
NRAS	3	4%	1	1%	7	4%	11	2.6%
PIK3CA	13	16%	28	19%	35	18%	76	17.8%
PTEN			3	2%	5	3%	8	1.9%
RB1					2	1%	2	0.5%
RET			1	1%		0%	1	0.2%
TP53	17	20%	20	13%	36	18%	73	17.1%
*no mutation* *	*33*	*40%*	*32*	*21%*	*44*	*23%*	*109*	*26%*

* No mutations were identified using the assays included in OncoMap

Overall, fewer tumors from Asian patients (60%) harbored one or more mutations queried by OncoMap, when compared to both Black patients (79%, P = 0.011) and White patients (77%, P = 0.015). Single mutations were found in 31% of tumors from Asians, 47% from Blacks and 39% from Whites, and multiple mutations in 29%, 32% and 38% respectively.

**Figure 2:**
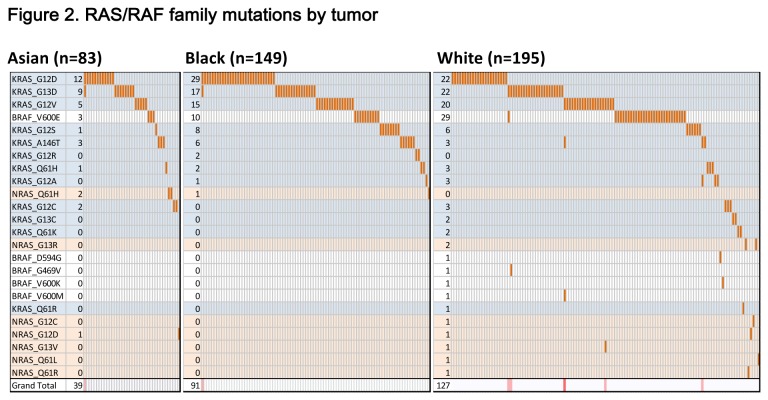
RAS/RAF family mutations by tumor and gene. A summary chart of all RAS/RAF mutations in each patient group displays genes across in rows and tumors down in columns. The bottom row of each represents the total number of mutations per tumor as a heat map (0-5 mutations). Chart A displays Asian patients, chart B, Black patients and chart C, White patients.

In colorectal cancers from White patients, we identified significantly more mutations in *BRAF* (17%) than in either the Asian (4%, P = 0.004) or Black (7%, P = 0.014) cohorts (Table 4). This was largely driven by a preponderance of *BRAF* V600 mutations in the White cohort. We found *BRAF* mutations to be more common in women, in older patients, and in the proximal colon, consistent with other reports [17,18,19]. Patients with *BRAF* mutant tumors had an average age of 72, compared to the average age of 65 for the entire cohort, and the cancer was located more commonly in the proximal colon (65% compared with 49% for complete cohort). Patients with *BRAF* mutant tumors were largely Stages III and IV. A higher frequency of *KRAS* mutations was observed in tumors from Black patients (Asian, 39%; Black, 53%; White, 44%) driven specifically by a prevalence of *KRAS* G12D mutations (Figure 2); however, this did not reach statistical significance.

**Table 4:** Patient characteristics for wild type and mutant BRAF.

**Features**		**wt-BRAF**		**m-BRAF**		**Total**	
**Age at diagnosis (mean, st dev)**		64.7	13.9	72.3	11.2	65.5	13.9
**Gender**							
	*Female*	194	51%	30	65%	224	52%
	*Male*	182	48%	15	33%	197	46%
	*unknown*	5	1%	1	2%	6	1%
		**381**	**100%**	**46**	**100%**	**427**	**100%**
**Race**							
	*Asian*	80	21%	3	5%	83	19%
	*Black*	139	36%	10	22%	149	35%
	*White*	163	43%	33	73%	195	46%
		**381**	**100%**	**46**	**100%**	**427**	**100%**
**Stage**							
	*I*	14	4%	0	0%	14	3%
	*II*	73	19%	2	5%	75	18%
	*III*	208	55%	28	65%	236	55%
	*IV*	51	13%	9	21%	60	14%
	*unknown*	38	10%	4	9%	42	10%
		**384**	**101%**	**43**	**100%**	**427**	**100%**
**Location**							
	*Distal*	102	34%	9	21%	111	26%
	*Proximal*	180	60%	28	65%	208	49%
	*Rectal*	66	22%	3	7%	69	16%
	*Colon, unspecified*	36	12%	3	7%	39	9%
		**384**	**127%**	**43**	**100%**	**427**	**100%**

Six tumors harbored more than one mutation in the *RAS/RAF* family (*KRAS*, NRAS, HRAS and *BRAF*), with all of them having at least one *KRAS* mutation (Figure 2). Of these, two tumors (1 from an Asian patient, 1 from a Black patient) had mutations in both *KRAS* G12D and G13D, which have been previously observed [19]. There were 2 instances of *KRAS* A146T, each of which co-occurred with a G12 mutation. *KRAS* A146T mutations have been seen in both colorectal cancers and colonic adenomas [20], and were associated with a more favorable outcome. Furthermore, some A146T mutations have been associated with copy number amplification of the *KRAS* locus.

We found 3 instances of co-occurring *BRAF* and *KRAS* mutations, all in tumors from White patients that harbored 4 or more mutations. Co-occurring were *BRAF* V600E and *KRAS* G13D, *BRAF* V600M and *KRAS* G12V, and *BRAF* G469V with both *KRAS* A146T and *KRAS* G13D. While rare, co-occurrences have been reported [19,21], and may represent different populations of cells within the tumor. An evaluation of the *KRAS* and *BRAF* allele spectral peak heights in the raw genotyping data shows that in one case, the mutant allele fractions of the genes were different, indicating that these may not be present at the same frequency in the specimen.

We identified *APC* mutations in 14% of tumors from Asian patients, 17% from Black patients and 19% from White patients. *APC* mutations, as expected for early events in colorectal cancer tumorigenesis, primarily co-occur with other mutations, most commonly with *KRAS*, *PIK3CA* and/or *TP53*. Because *APC* is a large tumor suppressor gene and OncoMap only assays for known recurrent variants, the number of mutations identified by genotyping likely under-represents the true prevalence of *APC* mutations in these cohorts.

*PIK3CA* mutations nearly always co-occurred with other mutations (92% of cases) with the most common partners found to be *RAS/RAF* family members (*KRAS*, NRAS and *BRAF*). *PIK3CA* mutations tend to cluster in exons 9 and 20 with exon 20 kinase domain mutations more common in breast cancer [22] and exon 9 helical domain mutations more common in colorectal cancer. As expected, we saw a predominance of helical domain mutations in our cohorts (Asian, 57%; Black, 59%; White, 65%). A few tumors harbored two distinct *PIK3CA* mutations; in one instance R88Q and C420R (p85 and C2 domains), and in another C420R and H1047R (C2 and kinase domains). A third sample (Asian) also had two helical domain *PIK3CA* mutations (E542K and E545K) along with a *KRAS* and a *TP53* mutation. While double *PIK3CA* mutants have been previously reported [22], their significance is unclear.

## Discussion

Using the OncoMap platform, we assayed for 385 known and clinically relevant somatic mutations in colorectal cancers from 427 Asian, Black and White patients to evaluate possible differences in the frequency of somatic mutations in colorectal cancer. In our study we found the mutation rate for cancers from Asians to be significantly lower than either the Black or White cohort. We found significant differences in the frequency of *BRAF* mutations with *BRAF* V600E mutations occurring most frequently in White patients. We find *BRAF* mutations to be more common in cancers from White patients, women, older patients, and in the proximal colon consistent with other reports [23]. In addition, while not significant using the more stringent criteria (Bonferroni correction), we found an enrichment of *KRAS* mutations in the Black cohort.

While the differences in *BRAF* mutation frequency between White, Black and Asian cohorts in colorectal cancer have not been previously reported, differences between other population groups have been noted. For example, in a recent study, Rozek et al [18] found the *BRAF* V600E mutation to be more common in persons of Ashkenazi Jewish descent and less common in those of Arab descent. Furthermore, English et al [24] found Australians of Anglo-Celtic descent to have a higher incidence of colorectal cancer and significantly more *BRAF* V600E mutations than those of Southern European descent. These findings demonstrate again that the frequency of important oncogenic mutations can vary in populations of different ancestral backgrounds. Whether and to what degree these variants have an impact on incidence, response to treatment or survival is yet to be determined.

The differences in the rates of *BRAF* mutation may reflect differences in the underlying etiology of genomic instability across these populations. *BRAF* mutations occur preferentially in the ascending or proximal colon in precursor lesions referred to as serrated adenomas. In the setting of *BRAF* mutation, these adenomas generally progress to cancer through an acquired DNA mismatch repair deficit caused by hypermethylation of *MLH1* in the setting of the CpG island methylator phenotype (CIMP) [25]. Thus these tumors tend to be characterized by microsatellite instability (MSI) and have high rates of somatic mutations, especially small insertions and deletions at small repeat regions. *KRAS* mutations, by contrast, occur more often in colorectal cancers that harbor chromosomal instability, the more common pattern of genomic aberrations in sporadic colorectal cancer.

The lower rates of *BRAF* mutant colorectal cancer seen in the Asian cohort could thus indicate that *BRAF* mutations themselves may be less common in this group. Alternatively, it is possible that there is a reduced propensity for pathologic DNA hypermethylation in the Asian population studied, thus reducing the ability of *BRAF* mutant precursor lesions to progress towards cancer. We did not have sufficient genomic material to pursue MSI testing or methylation testing for all specimens, and thus we were not able to directly measure if overall rates of CIMP-positive tumors were lower in the Asian population. However, for 62 of 83 Asian cancers assayed for MSI, only 3% (2/62) were MSI-high. This rate is lower than the normally observed rate (10-15%) in sporadic colorectal cancers, and consistent with a hypothesis that tumors driven by these mechanisms may be less common in the Asian population. Further work integrating more comprehensive genomic information with MSI assays and methylation status should clarify this.

The V600E mutation is an acquired activating mutation that results in constitutively active *BRAF* kinase activity leading to activation of the *MAPK* pathway. In melanoma, approximately 40% of patients have *BRAF* mutations of which 69% are V600E. The V600E has also been found in benign nevi [26], suggesting that V600 mutants may be early or precursor events. Similarly, *BRAF* mutations have been found in colorectal adenomas suggesting an early event in tumorgenesis [27]. Ogino et al [28], looking at clinical trial results (CALGB 89803) from 1264 stage III colorectal cancer patients, clarified predictive and prognostic effects of *BRAF* mutations and found *BRAF* mutants to be associated with worse outcome. Although the frequency of *BRAF* mutations is low (Asian, 4%; Black, 7%; White, 17%), compared to *KRAS* (Asian, 39%; Black, 53%; White, 43%), *BRAF* may be an important target for patients whose tumors harbor mutations.

Our finding of a lower mutation rate in our Asian cohort is of interest. The Asian cohort is comprised of significantly more lower stage patients and the cancers are predominantly distal (Asian, 40%; Black, 25%; White, 21%) rather than proximal. There does not appear to be an association between cancer location and mutation rate. If we look at mutation rates by cancer location we find that in the Asian cohort 48% of patients with distal cancers have no mutations compared to 19% of the Black and 22% of the White cohort. Of the Asian patients with distal cancers with mutations, there are 23 mutations in 17 patients (1.35 mutations per patient), less than either the Black or White cohorts at 1.63 and 1.69 mutations per patient, respectively. However, the average stage for distal, proximal and rectal cancers is about the same. Moreover, while this finding may truly represent a lower mutation rate for the Asian cohort, it also may be that the spectrum of mutations in this population is not well represented in OncoMap.

The rising incidence of colorectal cancer in many Asian countries in the last few decades is marked [10,11,29] and incidence in Korea has now surpassed the rate for Asian Americans. Rates of mortality for Asian Americans, while considerably lower than either Blacks or Whites, have been steadily decreasing, likely due to the institution of screening as a standard medical test. While the drivers behind this complex picture are numerous, there is some evidence that shift from a more traditional Asian diet to a Western style diet containing more processed and red meats and alcohol [10,11], may be a contributor.

The differences in the location of the cancers across our cohorts are striking and significant with White patients having more proximal cancers and Asians having more distal cancers. It has been previously observed that distal cancers are more common in the Korean [12] population and we observe this difference in both our Korean cohort and our Asian American cohort. Looking at tumor stage, we find all groups to have stage III cancers most frequently but stage distribution is otherwise diverse. There were more stage II cancers in the Asian group and the fewest in the White group (Asian, 34%; Black, 18%; White, 11%), and more stage IV cancers in the White group and the least in the Asian (Asian, 5%; Black, 12%; White, 19%). Looking at the average stage for each group, we find Whites to have the highest average stage (Asian, 2.41; Black, 2.85; White, 3.09).

*KRAS* mutations are well known in numerous cancers, located most commonly at exon 2 amino acids G12 and G13, two adjacent amino acids located near the catalytic site, and have been shown to result in constitutive activation of the *MAPK* signaling pathway. The prevalence of *KRAS* mutations in the Black group is driven by the *KRAS* G12D, however, the significance of *KRAS* G12D enrichment is unclear. This observation has been previously reported by Sylvester et al [19], however, they noted a higher frequency of G13 mutations in colon cancers from Black patients compared to the White, which we did not observe.

While there is some evidence that *KRAS* exon 2 mutations may be associated with a worse prognosis [20], and that G13 mutations may result in a less aggressive cancer, the evidence regarding the different effects of specific G12 or G13 variants is sparse. There are a considerable number of possible nucleotide changes at G12 and 13 resulting in different mutations, and each of these may produce altered downstream activity. A 2001 study by Andreyev et al [30] found that in colorectal cancer, of all the codon 12 and 13 mutations, only GGT to GTT transversions, resulting in G12V, were associated with worse survival. In contrast, in pancreatic cancer, which has the highest incidence of *KRAS* mutations of any cancer, 2 small studies [31,32] showed evidence that patients with G12V (GTT) mutations have longer median survival and those with G12D (GAT) have shorter. A recent and intriguing finding by Garassino et al [33], showed in NSCLC cell lines, that the G12D mutant had increased sensitivity to sorafenib (a *MAP* Kinase pathway inhibitor) compared to G12V and G12C, suggesting that differences at the nucleotide level may have clinical implications.

However, Ogino et al [28], in looking at *KRAS* mutations in the CALGB 89803 trial, found no significant effect on survival or disease progression for *KRAS* mutant colorectal cancer patients. Except for the predictive significance for efficacy of anti-*EGFR* monoclonal antibodies in the setting of metastasis for *EGFR*-expressing tumors [13], *KRAS* mutations appear to have no effect on outcome.

**Figure 3:**
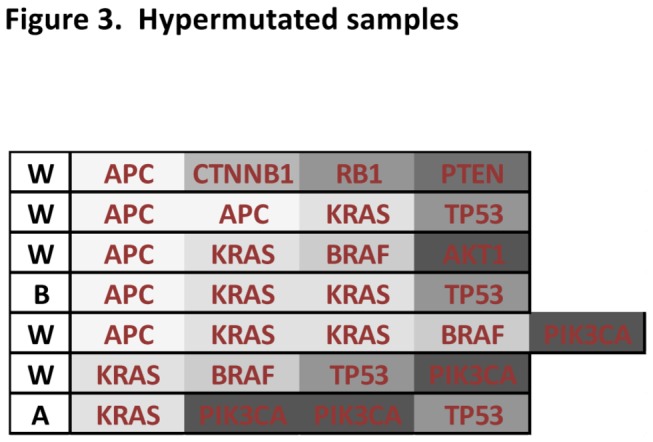
Hyper-mutated samples. Represented here are the genes mutated in the 7 patient samples that each have four or more mutations. A, W and B represent tumors from Asian, Black or White tumors, respectively.

Colorectal cancer is generally considered to progress from adenoma to carcinoma, with *APC* and *KRAS* mutations occurring early [34]; *APC* mutations in the normal epithelium and *KRAS* somewhere along the transition from small to medium size adenoma [34]. *TP53* mutations are late events. We have 7 patients each with more than 3 mutations (Figure 3), and when we look at these 7 samples, we can clearly see evidence of the generally accepted pathway of colorectal cancer progression: *APC*, as the first event, is found in 5/7; *KRAS*, a later event, in 6/7; and *TP53*, the last step to a carcinoma, is found in half of the samples.

We found the mutation rates of oncogenes to be at the expected frequencies [16]; however in tumor suppressor genes, our frequencies were considerably below what is expected. This is a limitation of the OncoMap platform in that it assays for known mutations and, while mutations in oncogenes tend to occur in the same locations, mutations in tumor suppressors occur more randomly across the gene. Next generation sequencing will be able to overcome most of the shortcomings inherent in OncoMap.

Genomic variations not included in OncoMap may yet explain some of the differences in our patient groups, for example, mutations in regions not covered by OncoMap, copy number or gene expression alterations, or epigenetic alterations. Further research should focus on an expanded set of genomic alterations, and integration of these data with MSI testing and/or assessment of CIMP in carefully controlled studies with extensive patient annotations where staging, diagnosis and treatment are equal.

In summary, we have identified differences in the somatic mutation frequency of known cancer genes in colorectal cancers from Asian, Black and White patients. These data argue in favor of using a genomics-driven precision medicine approach in order to elucidate the different ancestral determinants harbored by patient populations and thus, to more precisely and effectively treat colorectal cancer.
